# Antiproliferative effects of *Trigonostemon xyphophyllorides* on renal cell carcinoma via the PI3K/AKT pathway

**DOI:** 10.3389/fphar.2025.1594461

**Published:** 2025-11-07

**Authors:** Andong Wang, Yuru Yang, Tingting Chen, Jinyan He, Bai Ling, Xiaotian Cheng

**Affiliations:** 1 School of Pharmacy, Nantong University, Nantong, Jiangsu, China; 2 Department of Pharmacy, Yancheng Clinical College of Xuzhou Medical University and First People's Hospital of Yancheng, Yancheng, Jiangsu, China

**Keywords:** *Trigonostemon xyphophyllorides*, renal cell cancer, PI3K-Akt pathway, signaling pathway analysis, transcription analysis

## Abstract

**Background:**

One of the most well-known and deadly types of cancer is renal cell carcinoma (RCC). Not much research has been done on *Trigonostemon xyphophyllorides* (TX), an untested folk cure for cancer.

**Methods:**

UPLC-Q-TOF-MS/MS was used to systematically identify the components of TX. ACHN cell lines were used to assess TX’s pharmacological studies. Next, complexes with compound and gene will be included based on molecular docking and molecular dynamics simulations. In the end, signaling pathway analysis were used to elucidate the intricate mechanisms.

**Results:**

Analysis using UPLC-Q-TOF-MS/MS identified 47 major compounds in TX. *In vitro* experiments demonstrated that the TX extract was non-toxic to normal HK-2 cells but exhibited significant anti-proliferative effects on renal cancer ACHN cells, including inhibition of colony formation, suppression of cell migration, and anti-apoptotic properties. Transcriptomic analysis revealed that the anti-proliferative activity of TX was mediated through the PI3K-AKT signaling pathway. Subsequent validation was conducted using molecular docking, molecular dynamics simulation, qRT-PCR, and Western blotting techniques.

**Conclusion:**

This study utilized *in vitro* experimental validation techniques to establish that TX exerts its anti-proliferative effects by activating the PI3K-AKT signaling pathway. These findings elucidate the mechanism of TX action and provide a scientific basis for its prospective contemporary utilization.

## 1 Introduction

Renal cell carcinoma (RCC) represents about 4.1% of newly diagnosed malignancies, typically occurring at a median age of 64 years. It is a highly lethal cancer, exacerbated by a diet rich in sugar and fat (Rosiello et al., 2021). Due to resistance to chemotherapy and radiotherapy, radical surgery is the primary treatment, albeit with limited efficacy in advanced or metastatic cases (Campbell et al., 2021). Due to resistance to chemotherapy and radiotherapy, radical surgery is the primary treatment, albeit with limited efficacy in advanced or metastatic cases (Chakraborty et al., 2021). Therefore, it is necessary to find a new, safe and reliable treatment plan.

Transcriptomic analysis, integrating traditional pharmacology and network biology, enables target prediction by comprehensively integrating drug and disease information. For instance, transcriptomic analysis predicted 15 core genes as common targets between tanshinone, a key component of *Salvia miltiorrhiza*, and RCC. Kyoto Encyclopedia of Genes and Genomes (KEGG) analysis revealed anticancer effects through positive regulation of programmed cell death and apoptosis (Li K. et al., 2024). Similarly, oxymatrine, a main component of *Sophora flavescens*, was predicted to activate the PI3K/Akt pathway for therapeutic purposes (Chen et al., 2022). Thus, transcriptomic analysis offers a scientific approach to predict drug mechanisms.

*Trigonostemon*, a genus within the Euphorbiaceae family, comprises tropical and subtropical plants, with over 50 species found in tropical Asia from New Guinea to India and Sri Lanka. In traditional medicine, *Trigonostemon xyphophyllorides* (Croizat) L. K. Dai and T. L. Wu (TX) roots in Thailand are used for treating food poisoning and snake bites, while in China, the bark is employed for asthma-related conditions (Song et al., 2021). Modern separation techniques and frontier pharmacology research have identified diverse active components in *Trigonostemon* plants, including diterpenoids, alkaloids, coumarins, lignans, sesquiterpenoids, triterpenoids, flavonoids, and polyphenols. Notably, diterpenoids exhibit antiviral, antitumor, antibacterial, anti-inflammatory, and insecticidal properties. For instance, trigohowimine A, an abietane diterpenoid from this genus, demonstrates anti-proliferative effects on various tumor cell lines. Another compound, trigoflavidus A, exhibits significant anti-proliferative activity against renal cancer cells (Ban et al., 2022; Ban et al., 2020; Li et al., 2023). While literature reports on this genus, particularly its diterpenoid components, highlight their efficacy against renal cancer, studies often lack in-depth mechanistic exploration beyond IC_50_ anti-proliferation results. Therefore, this study analyzed chemical components of TX using UPLC-Q-TOF-MS/MS, predicted potential mechanism through transcriptomic analysis, and confirmed its activation of the PI3K-AKT signaling pathway for treating RCC through *in vitro* cell experiments.

## 2 Methods and materials

### 2.1 Plant material

In this study, the twig parts of *Trigonostemon xyphophyllorides* were collected in September 2023 from Haikou, Hainan province, China. The plant material was authenticated by Prof. Chen (Department of Pharmacy, Nantong University, Nantong 226,001, China). A voucher specimen (No. 2023026) has been deposited in the School of Pharmacy, Nantong University, Nantong, Jiangsu province, China.

### 2.2 Extraction of TX

In a reflux setting, the TX was collected and extracted three times, for 2 hours each time, using 95% EtOH. A vacuum rotary evaporator was used to evaporate the solvent in order to produce the 95% ethanolic extracts. A three-fold bed volume of elution mobile phase (methanol:water) was used to elute the silica gel chromatographic column. Using a YMG C18 (20 × 250 mm) column and gradient elution mobile phase acetonitrile at a flow rate of 2 mL/min for 0 min (20%), 2 min (30%), 10 min (60%), and 35 min (90%), UPLC-Q-TOF-MS/MS was used to systematically identify the components of TX (Zhao et al., 2023).

### 2.3 Cell lines and cell viability

In a humidified environment with 5% CO_2_ at 37 °C, the ACHN and HK-2 cells (Shanghai Institutes for Biological Sciences, Chinese Academy of Sciences) were cultivated in RPMI 1640 media supplemented with 10% fetal bovine serum (FBS), 1% streptomycin, and penicillin. STR profiling was used in our work to validate the cell lines. In 96-well microtiter plates, cells were plated at a density of 5 × 10^3^/well and exposed to different TX doses (4, 8, 12, 16, and 20 μg/mL). The CCK-8 assay was used to evaluate cell viability after treatment. A microplate reader (Synergy HT, Bio-Tek, USA) was used to measure absorbance at 450 nm (Schmidt et al., 2021).

### 2.4 Colony formation and wound healing assay

A colony formation experiment was carried out to evaluate the cells’ capacity for long-term proliferation. For 10 days, 400 monolayer cells per well were cultivated in 6-well plates. Following a 15-min methanol fixation period, cells were stained with GIMSA for 30 min. After taking pictures of the colonies, ImageJ software was used to count how many there were (Yang et al., 2023). After being planted in 6-well plates, the monolayer cells were cultured in the serum-free media for the entire night. A sterile 200 µL pipette tip was then used to scratch wound the cell. Cells were cultured in the serum-free medium for a further 48 h after being washed with PBS. A Nikon light microscope (Tokyo, Japan) was used to capture images of random fields. The percentage of wound closure was calculated using the formula below: percent wound closure (%) = [1−(Lt/L0)]×100, where L0 is the initial scratch width and Lt is the scratch width at time t (Al-Samydai et al., 2022).

### 2.5 Annexin V-FITC/PI apoptosis assay

After serum fasting, ACHN were plated in a 6-well plate at a density of 1 × 10^5^ cells per well and treated with TX for 48 h. The cells were then taken out and put in a binding buffer. After applying a combination solution of Annexin V-FITC (5 μL) and PI (5 μL) to the 400 μL cell suspension, it was incubated for 15 min in the dark. The samples were examined using flow cytometry, and the FlowJo 7.6 software was used to evaluate the data that was obtained (Zhu et al., 2022).

### 2.6 Dual staining using acridine orange (AO) and ethidium bromide (EB)

In short, the cells were seeded at a density of 2 × 10^2^ cells per well in a 24-well plate, treated with TX at various concentrations, and then incubated for 24 h at 37 °C with 5% CO_2_. The cells were stained with 10 µL of staining dye combination (10 mg/mL AO and 10 mg/ml EB) and incubated for 15 min following incubation and PBS (phosphate buffered saline) wash. A fluorescent microscope was used to examine the cells’ fluorescence (Chandrasekaran et al., 2023).

### 2.7 Transcription analysis

The raw sequencing data underwent extensive preprocessing to produce clean reads suitable for further investigation. Initially, SOAPnuke (version 1.6.5) was used to filter away reads with a low-quality base ratio (quality score≤15) over 40%, reads containing more than 1% of unknown bases, and reads contaminated by adaptor sequences. The clean reads were then stored in the FASTQ format. The cleaned data was then mapped to the created unique gene sequences using Bowtie2 (version 2.4.5). Following mapping, the gene expression levels were precisely quantified using RSEM (version 1.3.1). The genes have been annotated using public databases such as KEGG and GO. This annotation procedure provided important information about the biological roles of the genes. DESeq2 was used to identify genes that are differentially expressed between groups with fold change (FC) > 2 or <0.5. This was done in order to eliminate differentially expressed genes (DEGs) with fragments per kilobase of transcript per million fragments mapped (FPKM) values less than 1 in both comparison groups and to ensure that the detection rate of DEGs is greater than or equal to 66.6% in at least one group. The KEGG and GO annotations were used to functionally classify the DEGs (Rodríguez-Molina et al., 2023).

### 2.8 Construction of the PPI network

For study of PPI, the putative common targets were imported into the STRING database (Szklarczyk et al., 2019). To ensure the authenticity and accuracy of the data, we set a confidence level of 0.7 as an analysis criterion. The data was then processed in Cytoscape 3.10.2, where the degree value correlated with node size and the combination score were used to determine edge thickness. This led to the creation of a network diagram that illustrates the relationships between common protein targets. Using node-based topological centrality measures, we were able to determine significant targets of drugs against RCC by using the median values of Degree, Betweenness Centrality, and Closeness Centrality. Each node’s degree indicates how many direct connections it has; the more degrees it has, the more effect it has. Using Cytoscape 3.10.2, the interaction networks were displayed.

### 2.9 KEGG pathway enrichment and GO function analysis of key targets

The obtained key targets were imported into the Sandbox database (golgi.sandbox.google.com). Humans were selected as the species, the minimum overlap value was set at 3, the *P* value cutoff was set at *P* < 0.05, and the minimum enrichment value was set at 1.5 for the KEGG pathway enrichment and GO function analysis of key targets. Bubble diagrams were utilized for the analysis of the KEGG pathway and GO function. The top 10 GO functions were subjected to a histogram analysis after the gene proportions were sorted from large to small.

### 2.10 Molecular docking

To molecularly dock compounds, use AutoDock software (Eberhardt et al., 2021). First, the protein PDB format file was downloaded from (https://www.rcsb.org/), and the ligands and water molecules were viewed using Discovery Studio. The ligands were opened in Chemdraw 3D, processed for energy reduction, and a file in MOL2 format was generated. A higher absolute value of binding free energy in docking indicates a more durable interaction between the ligand and receptor. Binding affinity (kcal/mol) is a measure of the free energy of interaction between drugs and target proteins.

### 2.11 Molecular dynamic simulations

After molecular docking is finished, the top four bond energies are selected for molecular dynamics simulation. Checking for protein chain breakage before to the simulation is essential. The first step in creating a complete protein file is to copy the protein’s FASTA format file content from (https://www.rcsb.org/). The protein is then pretreated using SPDBV, and any remaining information about the protein is filled in by hydrogenating it via https://proteins.plus/. The Gromaces version 2022 is the first to incorporate the amber 14sb force field, which is mostly utilized for molecular dynamics simulations of proteins, nucleic acids, and other biological macromolecules. Topology files for five molecular dynamics simulations can be found at https://www.bio2byte.be/acpype/submit/ (Yu et al., 2024). A limiting box was incorporated into the simulation subsequent to the addition of the TIP3P water model to the system. After positioning the edge of the box at least 1.2 nm away from the edge of the protein, water was introduced into the limiting box. After adding the sodium ion balancing system and finishing the protein charge balance, the salt solution’s concentration was brought down to 0.15 mol/L. After processing the electrostatic interactions with Particel-Mesh Ewald (PME), the steepest decent method was used for energy reduction, with a maximum of 50,000 steps. Both the van der Waals radius and the Coulomb force had cutoff distances of 1.4 nm. The MD simulation was then run for 250 ns at room temperature and room pressure after the system had been balanced using a canonical system and an isothermal and isopressure system (Cao et al., 2023). The cutoff value for the nonbonded interaction was determined to be 10 A. The V-rescale temperature coupling method was used to set the simulation temperature to 310 K, and the Berendsen method was used to adjust the pressure to 1 bar. The CPPTRAJ module was used to perform the root mean square deviation (RMSD) analysis. The MMPBSA.py script was used to determine the binding free energy of active chemicals and proteins using the molecular mechanics/generalized Born surface area (MM/GBSA) technique. One important measure of the effectiveness of pharmaceutical medications is the binding free energy; a lower value denotes a more stable compound (Zhou et al., 2022).

### 2.12 Western blotting

To ascertain the regulatory effects of TX and RCC on the nuclear accumulation of catenin proteins, we extracted nuclear proteins using the nuclear protein extraction kit (Solarbio, Beijing, China). RIPA buffer supplemented with 1 mM PMSF and protease inhibitors was used to lyse the cells. Following centrifugation, we measured the protein contents in the lysate using the BCA assay. After translocation, electrophoresis, and an hour-long blocking with 5% skim milk, proteins were incubated with primary antibodies in TBST (1:1000 dilution). Enhanced chemiluminescence was used to detect immunoreactive bands using a goat anti-rabbit IgG secondary antibody coupled with horseradish peroxidase (1:10,000 dilution in TBST). Using ImageJ software and standardization to a predetermined sample on an identical membrane, the band density was quantified (Crescitelli et al., 2021).

### 2.13 Quantitative real-time reverse transcription polymerase chain reaction (qRT-PCR)

Utilizing the Trizol reagent (Beyotime Biotechnology Co., China), total RNA was isolated from ovarian tissue. After using NanoDrop 2000 (Thermo Fisher, USA) to quantify the total RNA, the kit (Cwbio Century Biotechnology Co., China) reverse-transcribed the RNA into cDNA. A Quant Studio3 fluorescent quantitative PCR device was then used to amplify the cDNA in accordance with the SYBR kit (Cwbio Century Biotechnology Co., China). The PCR reaction was run at 95 °C for 10 min, then 40 cycles (95 °C for 15 s and 60 °C for 1 min). GAPDH served as an internal reference gene for the comparative approach (2^−ΔΔCt) utilized to calculate relative mRNA levels. Table 1 indicated the primer sequences that were utilized (Huada Gene Research Institute, China) (Yoo et al., 2023).

**TABLE 1:** Primer sequences for qPCR.

Primer name	Primer sequence (5′-3′)	Primer length/bp
PIK3CA	F CAC​GAC​CAT​CAT​CAG​GTG​AA	21
	R CCT​CAC​GGA​GGC​ATT​CTA​AAG​T	22
PIK3CD	F AAG​GAG​GAG​AAT​CAG​AGC​GTT	21
	R GAA​GAG​CGG​CTC​ATA​CTG​GG	20
TLR4	F AGA​CCT​GTC​CCT​GAA​CCC​TAT	21
	R CGA​TGG​ACT​TCT​AAA​CCA​GCC​A	22
AKT1	F AGC​GAC​GTG​GCT​ATT​GTG​AAG	21
	R GCC​ATC​ATT​CTT​GAG​GAG​GAA​GT	23
AKT2	F ACC​ACA​GTC​ATC​GAG​AGG​ACC	21
	R GGA​GCC​ACA​CTT​GTA​GTC​CA	20

### 2.14 Statistical analysis

The data was evaluated using the SPSS 22.0 program. The mean plus or minus standard deviation was used to display the data. Group comparisons were made using the Tukey post-test and one-way ANOVA; a p-value of less than 0.05 was deemed statistically significant.

## 3 Results

### 3.1 Isolation compounds of TX

**FIGURE 1:**
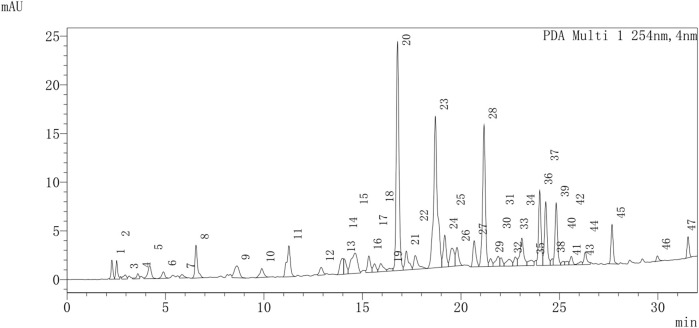
The chromatogram of TX.

UPLC-Q-TOF-MS/MS was used to provide a provisional identification of the chemicals of TX. The base peak chromatogram was shown in Figure 1, and a list of the likely compounds was provided in Table 2. The literature and the PubChem database were utilized to identify the compounds.

**TABLE 2:** The data of HPLC-Q-TOF-MS/MS from the twig parts of *Trigonostemon xyphophyllorides*.

Peak	Rt (min)	Molecular formula	Compound identified	Theoretical Mass (*m/z*)	Accurated Mass (*m/z)*	Error (ppm)	Ion species
1	1.64	C15H20O6	chavicol-1-*O-β-D*-glucopyranoside	296.1259	296.1259	0.1	(M + H)+
2	1.76	C20H20O8	spiciflorin	388.1157	388.1158	−0.25	(M-H)-
3	1.93	C29H26O15	lutescins B	614.1271	614.1271	0.1	(M-H)-
4	3.60	C15H14O4	murrayone	258.0892	258.0894	0.81	(M-H)-
5	4.89	C11H8N2O	1,2,3,4-tetrahydro-1-*oxo-β*-carboline	184.0637	184.0622	−7.82	(M + H)+
6	6.05	C12H8O4	xanthotoxin	216.0423	216.0435	5.61	(M + HCOO)-
7	6.26	C13H10O5	isoimpinellin	246.0528	246.0539	4.37	(M + CH3COO)-
8	6.40	C19H26O3	trigoheteric acid methyl ester	302.1882	302.1897	4.91	(M-H)-
9	8.11	C18H20O4	trigonostemon E	300.1362	300.1367	1.71	(M + H)+
10	8.14	C17H16O5	thrigonosomone D	300.0986	300.0998	−1.18	(M-H)-
11	8.82	C29H38O10	trigothysoid D	546.2476	546.2465	1.09	(M + H)+
12	9.02	C17H18O4	trigoxyphin M	286.1216	286.1205	1.12	(M + H)+
13	9.02	C17H24O5	3,4-dihydroxyallylbenzene-4-O-β-D-glucopyranoside	308.1624	308.1632	0.79	(M-H)-
14	9.06	C17H18O4	trigoxyphin G	286.1194	286.1205	−1.15	(M + HCOO)-
15	9.29	C36H40O10	trigonothyrin B	632.2598	632.2621	−2.37	(M + H)+
16	9.36	C16H18O4	trigoxyphin K	274.1189	274.1205	−1.59	(M + H)+
17	9.97	C16H18O3	trigoxyphin L	258.1262	258.1256	0.58	(M + H)+
18	10.07	C33H44O11	trigoheterin D	616.2863	616.2884	−2.07	(M + H)+
19	10.2	C32H26O6	neoboutomannin	506.1703	506.1729	−2.64	(M + H)+
20	10.80	C21H32O2	trigoheterophine D	316.2402	316.2372	−3.01	(M-H)-
21	10.83	C21H34O5	allo-pertusaric acid	366.2406	366.2427	2.07	(M-H)-
22	11.00	C34H30O6	trigohowilol D	534.2063	534.2042	2.1	(M + HCOO)-
23	11.05	C38H42O11	trigochinin C	674.2706	674.2727	−2.12	(M + H)+
24	11.06	C21H26O4	trigonochinene D	342.1835	342.1831	0.44	(M + Na)+
25	11.12	C42H48O14	trigochinin B	776.3027	776.3044	−1.67	(M + H)+
26	11.13	C42H50O15	trigohownin G	794.3154	794.315	0.41	(M + H)+
27	11.22	C18H22O2	trigonostemon A	270.1622	270.162	0.18	(M + H)+
28	11.24	C19H34O4	nephromopsinic acid	326.2457	326.248	2.26	(M-H)-
29	11.38	C35H32O10	trigoflavidol A	612.1967	612.1995	−2.84	(M-H)-
30	11.39	C34H36O10	trigothysois M	604.231	604.2308	0.11	(M + H)+
31	11.40	C33H28O6	trigohowilols C	520.1896	520.1886	1.01	(M + H)+
32	11.49	C40H44O12	trigoxyphin I	716.2832	716.2833	−0.09	(M + H)+
33	11.50	C25H24O6	trigoxyphin J	420.1582	420.1573	0.9	(M + Na)+
34	11.9	C40H46O13	trigochinin A	734.2921	734.2938	−1.73	(M + H)+
35	11.96	C34H30N2O8	lotthanongine	594.2002	594.2018	1.6	(M + CH3COO)-
36	11.97	C40H46O13	trigochinin D	734.293	734.2938	−0.85	(M + H)+
37	12.04	C34H40O10	trigonosins C	608.2603	608.2621	−1.87	(M + H)+
38	12.17	C34H38O9	trigonosin A	590.2526	590.2516	0.97	(M + H)+
39	12.51	C34H34O8	trigoxyphin H	570.2277	570.2254	2.38	(M + H)+
40	12.57	C36H46O4	trigonostemon H	542.3428	542.3396	3.19	(M-H)-
41	12.81	C43H56O13	rediocide E	780.3761	780.3721	3.97	(M + H)+
42	13.26	C20H32O4	mucorinic acid A	336.2301	336.2292	−0.86	(M + H)+
43	13.36	C34H38O10	trigonosin B	606.2474	606.2465	0.87	(M + H)+
44	13.49	C44H60O12	rediocide J	780.4083	780.4085	−0.16	(M-H)-
45	13.79	C44H58O13	rediocide A	794.3866	794.3877	−1.16	(M + H)+
46	14.54	C20H34O5	dihydropertusaric acid	354.2406	354.2375	−3.13	(M + Na)+
47	14.81	C36H42O11	trigonothyrin F	650.2729	650.2727	0.21	(M + H)+

### 3.2 Pharmacodynamic experiments verification *in vitro*

**FIGURE 2:**
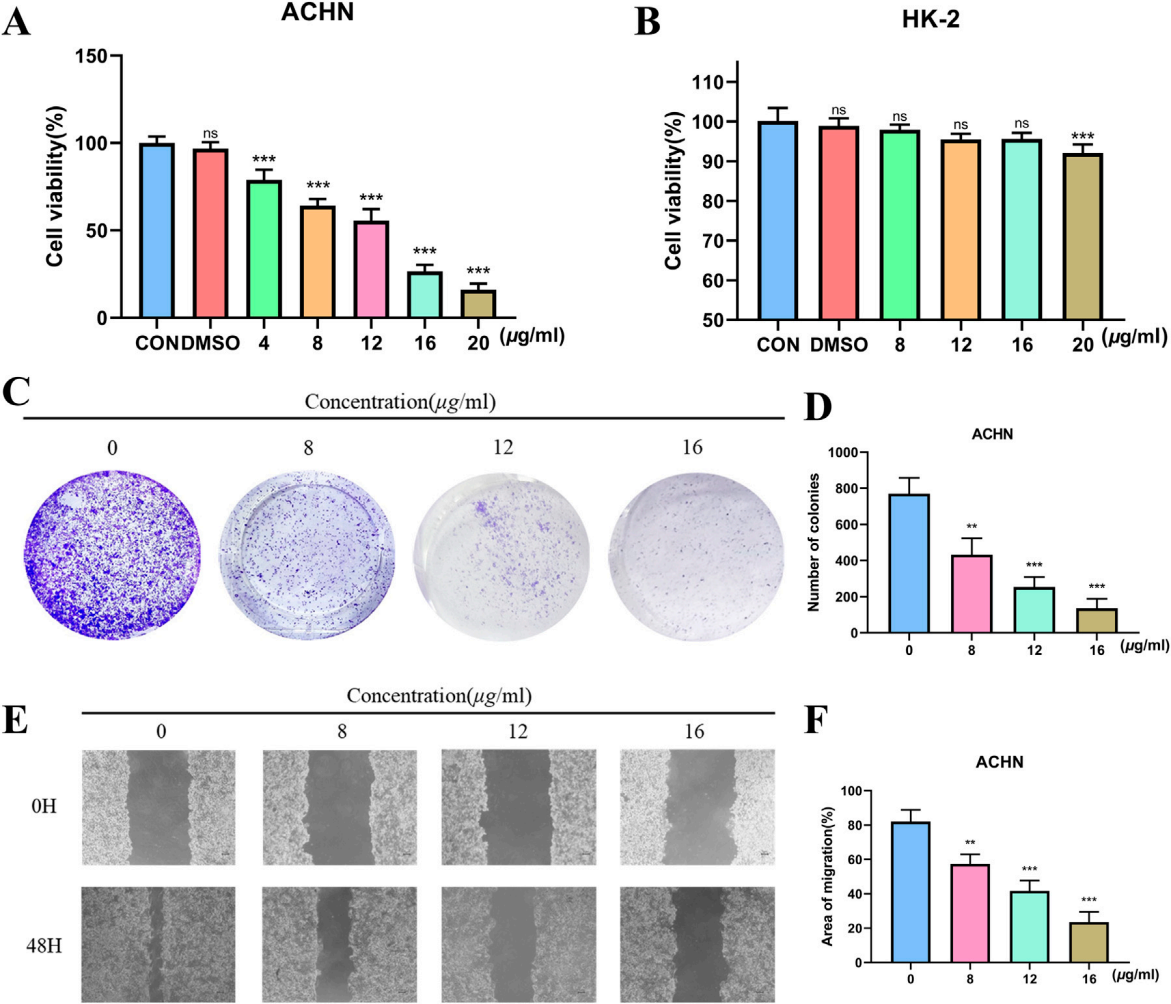
Antiproliferative activity of ACHN with varying concentrations of TX **(A)** cytotoxicity of HK-2 with varying concentrations of TX **(B)** colony formation assay with varying concentrations of TX **(C)** number of colonies with varying concentrations of TX **(D)** scratch wound healing and cell invasion assays with varying concentrations of TX **(E)** area of migration of scratch wound healing and cell invasion assays with varying concentrations of TX **(F)**. Note: * represents for vs. MOD group (*p* < 0.05), ** represents for vs. MOD group (*p* < 0.01), *** represents for vs. MOD group (*p* < 0.001), respectively.

We used a CCK-8 assay to look at how TX affected the viability of RCC (ACHN). TX dramatically decreased ACHN cell viability in a dose-dependent manner, as seen in Figure 2A. TX had strong antiproliferative action in the ACHN cell line, and its IC_50_ was 10.61 μg/mL. Furthermore, the cytotoxicity of treating cell lines (HK-2) with different TX doses for 24 h is shown in Figure 2B. The CCK-8 assay was used to evaluate the cells’ vitality. It proves that TX does not significantly harm HK-2 cells. Two significant malignant behaviors that occur during the metastatic process are tumor cell invasion and migration. It used a Transwell experiment to investigate how TX affected these behaviors in ACHN. The findings demonstrated that TX inhibits ACHN migration in a concentration-dependent manner by causing ACHN cells to migrate in smaller numbers from the upper chamber, which lacked serum, to the bottom chamber, which had serum (Figures 2C,D). In contrast to the control group (0 μg/mL TX), the control group showed considerable healing after 48 h, according to the wound-healing experiment. ACHN moved from the intact monolayer to the scratched area when TX concentrations rose to 8, 12, and 16 μg/mL. The migration rate decreased with increasing TX concentrations (Figures 2E,F), suggesting that TX markedly and dose-dependently prevented cancer cells from healing.

**FIGURE 3:**
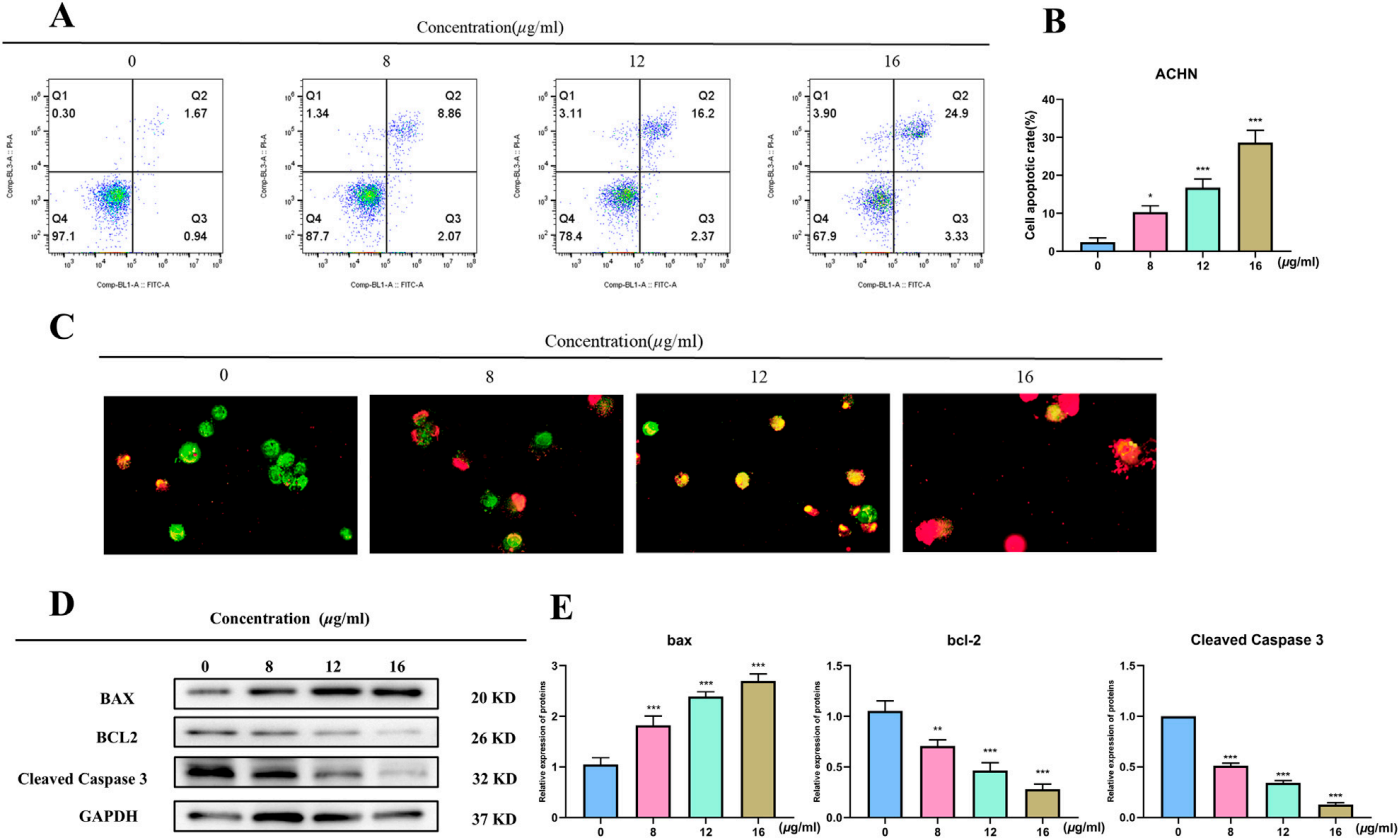
Flow cytometry of ACHN with varying concentrations of TX **(A)** cell apoptosis rate of flow cytometry **(B)** AO/EB staining images of TX-treated RCC cells **(C)** Western blot on Bcl2 and Bax with varying concentrations of TX **(D)** relative expression of BAX, Bcl-2 and Cleaved Caspase 3 protein **(E)**. Note: * represents for vs. MOD group (*p* < 0.05), ** represents for vs. MOD group (*p* < 0.01), *** represents for vs. MOD group (*p* < 0.001), respectively.

It used the Annexin V-FITC/PI double-staining technique in flow cytometry to detect apoptosis in order to examine if TX causes it. The proportion of apoptosis in ACHN rose in a dose-dependent way when compared to the control group, as seen in Figures 3A,B. In the 8 μg/mL TX-treated group, the percentage of apoptosis rose from 2.38 ± 1.17% in the control group to 10.30 ± 1.67%, 16.77 ± 2.27% in the 12 μg/mL TX-treated group, and 28.63 ± 3.22% in the 16 μg/mL TX-treated group, respectively (*P* < 0.05). It was demonstrated that ACHN cells were more responsive when TX was present in the same quantities. Dual AO/EB detection for necrosis and apoptosis staging, respectively, was used to analyze the modes of cell death. The current investigations revealed that late apoptotic cells displayed broken orange chromatin and early apoptotic cells had bright green nuclei with fragmented chromatin (Figure 3C). TX suppressed Bcl2 and Cleaved Caspase 3 and increased Bax protein expression in ACHN cells in a concentration-dependent manner, according to Western blot data, which confirmed the action of TX on Bcl2, Bax and Cleaved Caspase 3 (Figures 3D,E).

### 3.3 Transcription analysis

**FIGURE 4:**
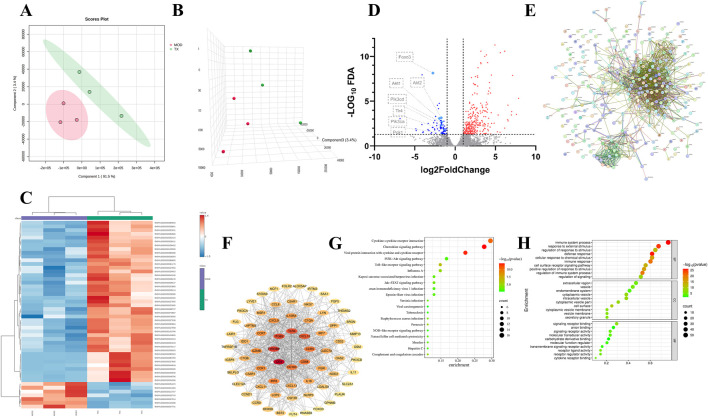
PCA 2D plots **(A)** PCA 3D plots **(B)** Top 50 genes thermodynamic diagram **(C)** volcano plot of serum samples between MOD and TX **(D)** PPI network **(E)** network of 69 major targets genes **(F)** Top 20 KEGG enrichment analysis **(G)** Top 10 GO enrichment analysis **(H)**. N = 3.

A total of 3,869 mRNA of ACHN cell treated with 16 μg/mL TX groups were identified by RNA-Seq with |log_2_(Fold Change)| > 1 and adj.*P* (adjusted *P* value) < 0.05. To differentiate between the groups and illustrate the transcription differences, PLS-DA (Figure 4A), PLS-DA-3D (Figure 4B), and heatmap (Figure 4C) analyses were performed based on the global features of the raw data. Between the MOD and TX groups, 303 genes were in up mode and 74 genes were in down mode (Figure 4D). The PI3K-AKT signaling pathway-related genes PCK1, PIK3CD, PIK3CA, AKT1, AKT2, and FOXO3 were all markedly reduced. The protein-protein interaction network that was produced from the STRING database illustrates the complex interactions that the proteins of these target genes displayed (Figure 4E). The degree centrality, closeness centrality, and betweenness centrality measurements of network topology were used to develop specific screening criteria: degree value > 7, BC ≥ 0.0019, and CC > 0.3272. This investigation identified 69 main targets, suggesting that these targets are significant network members (Figure 4F). To ascertain which pathways were affected by the 69 target genes, a KEGG enrichment analysis was conducted. A high enrichment of 83 KEGG pathways was discovered. These findings imply that the target genes are connected to many networks, including Cytokine-cytokine receptor interaction, Chemokine signaling pathway, Viral protein interaction with cytokine and cytokine receptor, PI3K-Akt signaling pathway, Toll-like receptor signaling pathway, Influenza A, Jak-STAT signaling pathway, Kaposi sarcoma-associated herpesvirus infection, Epstein-Barr virus infection, Human immunodeficiency virus 1 infection. Figure 4G showed the bubble plot with the top 20 KEGG pathways. The 69 target genes’ MF, CC, and BP were identified using GO enrichment analysis. 1491 highly enriched GO keywords were discovered. Figure 4H displayed a graphic representation of the top 10 terms. Integration of GO functional enrichment analysis reveals the activation of a cascade of cellular receptor pathways following drug administration, leading to the desired therapeutic outcomes.

### 3.4 Molecular docking

**FIGURE 5:**
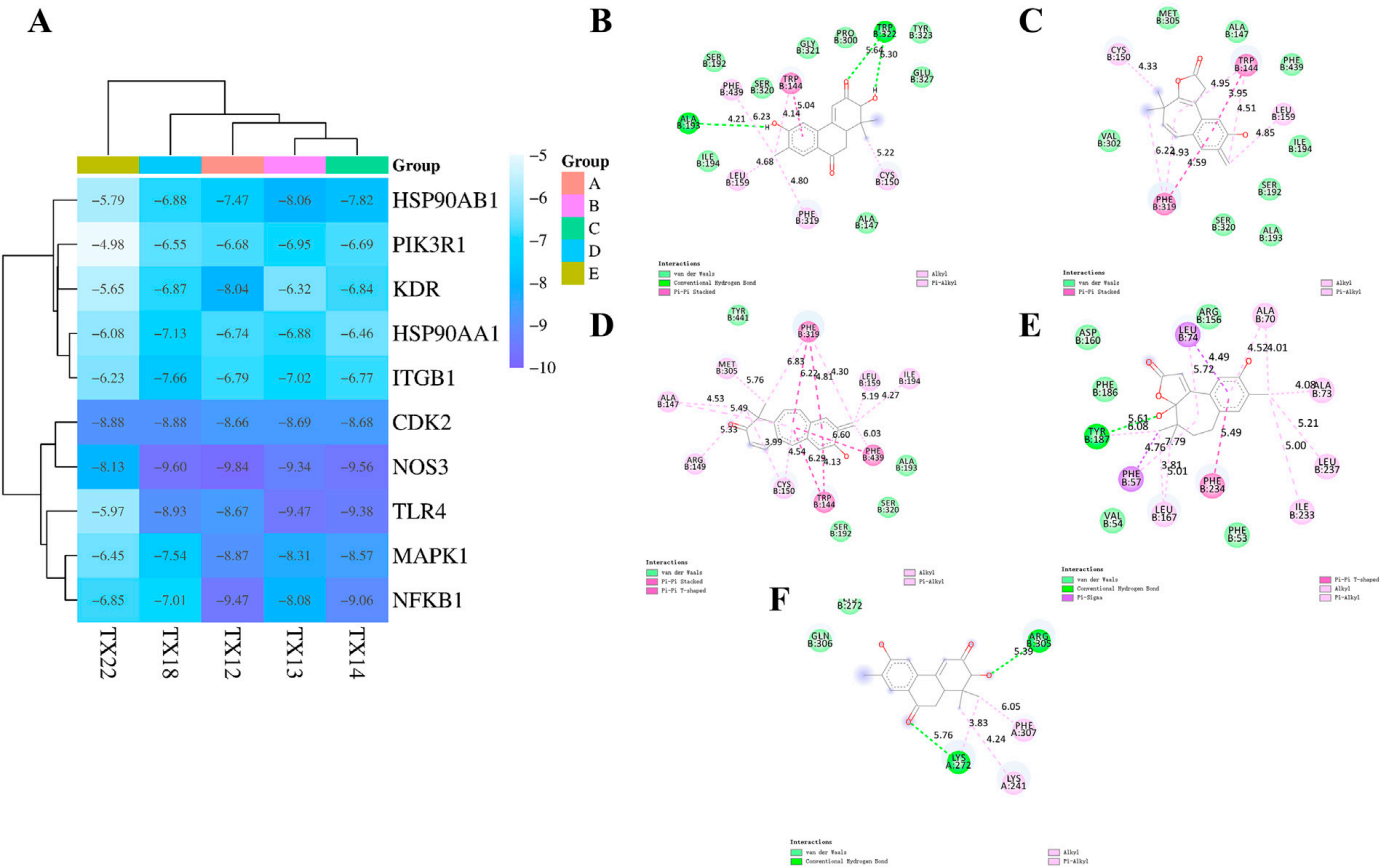
Docking heatmap of five compounds with ten genes **(A)** Docking effect drawing of 3EAH and TX12 **(B)** Docking effect drawing of 3EAH and TX14 **(C)** Docking effect drawing of 3EAH and TX18 **(D)** Docking effect drawing of 5UC8 and TX13 **(E)** Docking effect drawing of 8TKL and TX12 **(F)**.

Molecular docking is used to assess the binding interactions between small molecule drugs and potential targets to gain understanding of their therapeutic potential. Effective ligand binding to the receptor target protein is indicated by lower, more negative binding energies, which are indicative of stronger binding affinities. Using crystal structures from the PDB database, it describe the results of docking 47 chemicals with 10 key genes (3I5S for PIK3R1, 3T9K for ITGB1, 5UC8 for TLR4, 6SLG for MAPK1, 8TKL for NFKB1, 3PXQ for CDK2, 3EAH for NOS3, 5NJX for HSP90AA1, 5EW3 for KDR, and 3PRY for HSP90AB1) (Figure 5A). The top five binding energies were NOS3 and TX12/14/18, TLR4 and TX13, NFKB1 and TX12. The results showed that the docking scores of 23 compounds with ten major genes ranged from −4.98 kcal/mol to −9.84 kcal/mol. Lower docking scores indicate stronger binding affinity, scores less than −5.0 kcal/mol indicate potential binding, and scores more than −7.0 kcal/mol indicate stronger binding affinity.

Three types of interaction forces, van der Waals, hydrophobic, and hydrogen bonding, are evident in 3EAH and TX12. The generated van der Waals forces are ILE194, SER192, SER320, GLY321, PRO300, TYR323, GLU327, ALA47. Hydrophobic forces are PHE439 (6.23 Å), TRP144 (4.14 Å, 5.04 Å), LEU159 (4.68 Å), PHE319 (4.80 Å), CYS150 (5.22 Å). Amino acids that form hydrogen bonding forces are ALA193 (4.21 Å), TRP322 (5.64 Å, 6.30 Å) (Figure 5B). Two types of interaction forces are evident in 3EAH and TX14, namely van der Waals forces, hydrophobic forces. The generated van der Waals forces are VAL302, MET305, ALA147, PHE439, ILE194, SER192, ALA193, SER320. Hydrophobic forces are CYS150 (4.33 Å), TRP144 (4.95 Å, 3,95 Å, 4.51 Å), LEU159 (4.85 Å), PHE 319 (6.22 Å, 4.93 Å, 4.59 Å) (Figure 5C). Two types of interaction forces, van der Waals forces, and hydrophobic forces, are evident in 3EAH and TX18. The van der Waals forces generated are TYR441, ALA193, SER320, SER192. Hydrophobic forces are ALA147 (4.53 Å, 5.49 Å), MET305 (5.76 Å), PHE 319 (6.83 Å, 6.22 Å, 4.81 Å, 4.30 Å), LEU159 (5.19 Å), ILE194 (4.27 Å), PHE439 (6.60 Å), TRP144 (6.29 Å, 4.13 Å), CYS150 (3.99 Å, 4.54 Å), ARG149 (5.33 Å) (Figure 5D). Three types of interaction forces, van der Waals, hydrophobic and hydrogen bonding, are evident in 5UC8 and TX13. The van der Waals forces generated are ASP160, ARG156, PHE53, VAL54, PHE186. Hydrophobic forces are LEU74 (4.49 Å, 5.72 Å), ALA 70 (4.52 Å, 4.01 Å), ALA 73 (4.08 Å), LEU237 (5.21 Å), ILE233 (5.00 Å), PHE 234 (5.49 Å), LEU167 (3.81 Å, 5.01 Å), PHE57 (4.76 Å, 7.79 Å), ARG149 (5.33 Å), TYR187 (6.08 Å). The resulting hydrogen bonding force is TYR187 (4.76 Å) (Figure 5E). Three types of interaction forces are evident in 8TKL and TX12, namely van der Waals, hydrophobic and hydrogen bonding forces and others. The van der Waals forces generated are LYS272, GLN 306. Hydrogen bonding forces are ARG305 (5.39 Å), LYS 272 (5.76 Å). Hydrophobic forces generated are PHE307 (6.05 Å), LYS 241 (4.24 Å), LYS 272 (3.83 Å) (Figure 5F). It's interesting to note that NOS3 bonded well to every significant chemical. These findings suggest that NOS3 might target these substances to have anti-RCC effects.

### 3.5 Molecular dynamics simulations

Molecular dynamics simulation is the most efficient method for assessing the binding affinity of molecular drugs to key target proteins. In our molecular dynamics modeling approach, we selected four standard metrics to assess the protein-ligand binding strength: root mean square deviation (RMSD), root mean square fluctuation (RMSF), hydrogen bonding (H-Bond), and radius of gyration (RG). Notably, the evaluation does not consider the quantity of hydrogen bonds formed due to the absence of stable hydrogen bonding forces during the simulation.

**FIGURE 6:**
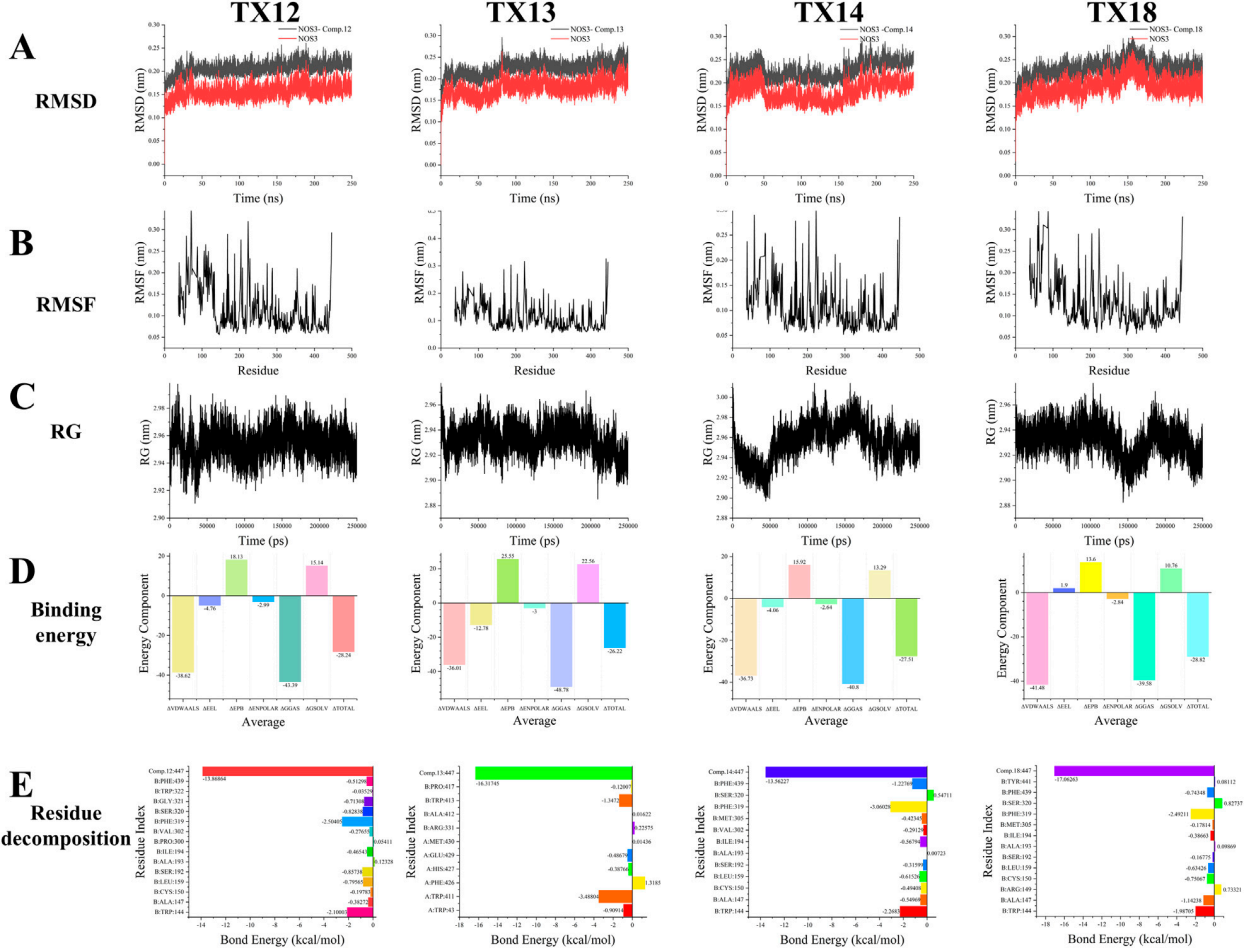
RMSD **(A)** RMSF **(B)** RG **(C)** Binding energy **(D)** Residue decomposition **(E)**.

RMSD of a protein indicates the deviation in position between its initial conformation and its conformation throughout the simulation. Monitoring the RMSD trends of protein-ligand complexes is critical for assessing simulation stability. For the NOS3-TX12 binding process, the RMSD trend remains consistently below 0.1 nm, suggesting a highly stable binding interaction. Similarly, the NOS3-TX13 binding exhibits a stable trend, with minimal fluctuations between 0 and 80 ns, indicating sustained stability. In the case of NOS3-TX14 binding, despite an initial significant fluctuation, stability is achieved between 0 and 50 ns, with subsequent fluctuations around 50 ns followed by stability from 55 to 155 ns, and a slight increase thereafter. This stable state persists until the end of the simulation. Furthermore, the NOS3-TX18 binding maintains stability from 0 to 130 ns, with notable fluctuations between 150 and 185 ns, leading to enhanced stability post-185 ns until the simulation’s completion (Figure 6A).

The flexibility and dynamic motion of protein amino acids can be quantified using RMSF, representing the average positional variance of atoms over time. In this study, RMSF was employed to assess the flexibility of protein amino acids and their correlation with active amino acids involved in ligand binding. Our analysis reveals that amino acid residues exhibiting higher flexibility during the binding of NOS3 to TX12 correspond to active binding sites crucial for binding stability, specifically residues 30, 58, 71, 168, 187, 204, 223, 274, 287, and 355. Notably, the terminal amino acid residue, displaying increased flexibility, predominantly contributes to the pronounced deformation post-amino acid 440. Similarly, upon NOS3 binding to TX13, protein amino acid residues exhibit enhanced flexible deformation. The key binding sites encompass residues 39, 58, 168, 187, 204, 223, 274, 287, and 355, which undergo significant deformation. Once again, the terminal amino acid residue, characterized by heightened flexibility, primarily drives the substantial deformation post-amino acid 440. The lack of protein alterations during binding with different ligands resulted in similar outcomes for NOS3 with TX14 and TX18, mirroring the findings mentioned earlier. These dynamic changes primarily stem from the unchanged nature of the proteins or their binding sites (Figure 6B).

RG of the protein complex serves as an indicator of structural tightness and can elucidate changes in the tightness or looseness of protein peptide chains over the simulation period. During the binding of NOS3 to TX12, the protein’s maximum RG remained consistently within 3 nm with fluctuations not exceeding 0.1 nm, indicating structural stability. Conversely, when NOS3 binds to TX13, the RG fluctuates within 0.1 nm, signifying well-maintained interactions that enhance protein stability. This observation underscores the exceptional stability of NOS3 throughout the simulation, likely contributing to its biological functionality and molecular interactions. In the case of TX14 binding, NOS3 exhibits RG variations within 0.1 nm, suggesting overall stability despite potential localized flexibility within certain regions of the protein structure. This suggests that the protein exhibits overall stability with a moderate level of flexibility to accommodate ligand binding or interactions with other molecules. The well-maintained connections contributing to the protein’s stability support the hypothesis that NOS3 remains highly stable throughout the simulation, with its biological functions closely associated with these interactions. Despite minor fluctuations in the radius of gyration, some proteins can maintain a specific conformation while performing their biological roles. However, during the binding of NOS3 to TX18, the protein’s radius of gyration fluctuated within a narrow range of 0.1 nm. This observation implies that the protein may not necessitate significant conformational changes to execute its function (Figure 6C).

The binding energy is the sum of ΔGGAS (a component of the binding energy) and ΔGSOLV (a component of the solvation energy). ΔGGAS represents the free energy change of the interaction in the system, with a negative value indicating spontaneous binding. On the other hand, ΔGSOLV reflects the energy change due to solvation effects, having a positive value that signifies a loss of energy for binding in the solvent. In NOS3 simulations with TX12, ΔGGAS is −43.39 kcal/mol, and ΔGSOLV is 15.14 kcal/mol. The total binding free energy (ΔTOTAL) is −28.24 kcal/mol, also indicating spontaneous ligand-receptor binding. Similarly, in simulations with TX13, ΔGGAS is −48.78 kcal/mol, ΔGSOLV is 22.56 kcal/mol, and ΔTOTAL is −26.22 kcal/mol, all pointing towards spontaneous binding. Moreover, simulations with TX14 reveal ΔGGAS as −40.8 kcal/mol, ΔGSOLV as 13.29 kcal/mol, and ΔTOTAL as −27.51 kcal/mol, all indicating spontaneous ligand-receptor binding. Similarly, in simulations with TX18, ΔGGAS is −39.58 kcal/mol, ΔGSOLV is 10.76 kcal/mol, and ΔTOTAL is −28.82 kcal/mol, all suggesting spontaneous ligand-receptor binding (Figure 6D).

Residue decomposition is utilized for analyzing energy contributions and identifying key residues. In simulations of NOS3 and TX12, B:TRP:144 has an average total of −2.1001 kcal/mol, with the predominant change stemming from van der Waals and electrostatic interactions. B:ALA:147 exhibits an average total of −0.3827 kcal/mol, with a minor contribution primarily attributed to van der Waals forces. B:CYS:150 shows an average total of −0.1978 kcal/mol, with a negligible contribution that is essentially neutralized by the combined energies. B:LEU:159 demonstrates an average total of −0.7956 kcal/mol, with the main contribution arising from van der Waals forces. B:SER:192 showcases an average total of −0.8573 kcal/mol, with a minor contribution predominantly from van der Waals interactions. In the case of B:SER:192, the average total is −0.3827 kcal/mol, with electrostatic and polar solvation energies offsetting each other, primarily due to van der Waals interactions. At B:ALA:193 and B:ILE:194, the average total energy is 0.1232 kcal/mol, with minimal variation, where the polar solvation energy slightly exceeds the van der Waals interaction. At B:ILE:194, the average total energy is −0.4654 kcal/mol, predominantly attributable to the van der Waals interaction. Moving to B:PRO:300 and B:VAL:302, the average total energy is −0.2765 kcal/mol, with an insignificant contribution, primarily from the van der Waals effect. At B:PHE:319, the average total energy is −2.5041 kcal/mol, primarily stemming from electrostatic and van der Waals interactions. For B:SER:320, the average total energy is −0.8283 kcal/mol, where electrostatic and polar solvation energies counterbalance, predominantly from van der Waals forces. At B:GLY:321, the average total energy is −0.7131 kcal/mol, with electrostatic and polar solvation energies nullifying each other, mainly due to van der Waals interactions. Lastly, at B:TRP:322, the average total energy is −0.0353 kcal/mol, with minimal overall change, as all energy components effectively cancel each other out. At position PHE:439, the average total energy is −0.5129 kcal/mol, primarily attributed to van der Waals forces, with electrostatic and polar solvent effects partially offsetting each other. Conversely, at position L:Comp.12:447, the average total energy is −13.8686 kcal/mol, with the most significant energy change arising from electrostatic interactions, followed by van der Waals forces and polar solvent effects. Specifically, the largest energy perturbation is due to electrostatic interactions, succeeded by van der Waals forces and polar solvation contributions. Notably, TRP144, PHE319, and L:Comp.12:447 were the most influential residues in terms of total energy changes, with respective values of −2.10, −2.50, and −13.87 kcal/mol. The predominant energy changes in most residues stemmed from van der Waals interactions, while electrostatic and polar solvation effects tended to counterbalance each other. In simulations involving NOS3 and TX13, residue A:TRP:43 exhibited an average total energy of −0.9091 kcal/mol, primarily driven by van der Waals interactions, partially counteracted by electrostatic and polar solvation contributions. A:TRP:411 has an average total energy of −3.4881 kcal/mol, primarily attributed to electrostatic interactions, followed by Van der Waals forces. A:PHE:426 exhibits an average total energy of 1.3185 kcal/mol, with electrostatic, Van der Waals, and polar solvation energies being counteracted by non-polar solvent interactions, resulting in a positive total change. A:HIS:427 shows an average total energy of −0.3877 kcal/mol, where the contributions of electrostatic, Van der Waals, and polar solvation energies nullify each other. A:GLU:429 demonstrates an average total energy of −0.4868 kcal/mol, with electrostatic and polar solvation energies offsetting each other, while the main contribution comes from Van der Waals forces. A:MET:430 displays an average total energy of 0.0144 kcal/mol, with minimal total change as all energies effectively cancel each other out. B:ARG. 331 has an average total energy of 0.2258 kcal/mol, where electrostatic and polar solvation energies balance each other out, resulting in a small total change slightly above zero. B:ALA: 412 exhibits an average total energy of 0.0162 kcal/mol, with minimal total change as all energies effectively nullify each other. B:TRP413 has an average total energy of −1.3472 kcal/mol, primarily driven by electrostatic and van der Waals forces, with a minor offset from polar solvation energies. B:pro:417 exhibits an average total energy of −0.1201 kcal/mol, with minimal overall change as the energies effectively counterbalance each other. L:Comp.13:447 displays the most significant energy alteration with an average total energy of −16.3174 kcal/mol, predominantly influenced by electrostatic interactions and polar solvation energy, followed by nonpolar solvent effects. Notably, TRP411, TRP413, and L:Comp.13:447 contribute the most to the total energy changes, with values of −3.49, −1.35, and −16.32 kcal/mol, respectively. Conversely, PHE426 exhibits a positive total energy change of 1.32 kcal/mol, suggesting a dominant nonpolar solvent interaction. The remaining residues show minor total energy changes, with electrostatic, van der Waals, and polar solvation energies largely offsetting each other. The considerable standard deviations and standard errors indicate substantial fluctuations in these energy values. In the NOS3 and TX14 simulations, B:TRP:144 has an average total energy of −2.2683 kcal/mol, primarily driven by van der Waals interactions and polar solvation energy contributions. At position B:ALA:147, the average total energy is −0.5497 kcal/mol, primarily attributed to van der Waals, electrostatic, and partially offset polar solvation interactions. At B:CYS:150, the average total energy is −0.4941 kcal/mol, with electrostatic, van der Waals, and polar solvation energies offsetting each other. Similarly, at B:ALA:150, the average total energy is −0.4941 kcal/mol, with comparable contributions from electrostatic, van der Waals, and polar solvation interactions. At B:LEU:159, the average total energy is −0.6153 kcal/mol, predominantly influenced by van der Waals interactions. Moving to B:SER:192, the average total energy is −0.3159 kcal/mol, with minimal impact as the various energy components effectively neutralize each other. Considering B:ALA:193, the average total energy is 0.0072 kcal/mol, indicating a near-zero net change as the energy terms cancel out. At B:ILE:194, the average total energy is −0.5679 kcal/mol, primarily driven by van der Waals interactions. At B:VAL:302, the average total energy is −0.2913 kcal/mol, with energy contributions being negligible and mutually compensatory. Lastly, at B:MET:305, the average total energy is −0.0072 kcal/mol, with minimal overall change as the various energy components cancel each other out. At residue B:MET:305, the average total energy change is −0.00722 kcal/mol, with a negligible overall change primarily resulting from the offsetting of various energy components such as van der Waals, electrostatic, and polar solvation energies. The total energy at this residue is −0.4234 kcal/mol, with a minor contribution mainly from van der Waals interactions, while electrostatic and polar solvation energies partially counteract each other. At residue B:PHE:319, the average total energy change is −3.0602 kcal/mol, primarily driven by electrostatic and van der Waals interactions. For residue B:SER. 320, the average total energy change is 0.5471 kcal/mol, showing a positive shift mainly attributed to polar solvation energy, with electrostatic and van der Waals effects partially balancing each other. At residue B:PHE:439, the average total energy change is −1.2277 kcal/mol, predominantly influenced by van der Waals interactions. Following this, residue L:Comp.14:447 exhibits the most substantial energy change at −13.5623 kcal/mol, primarily due to electrostatic interactions, followed by polar solvation energy, with a minor contribution from van der Waals forces. Overall, residues TRP144, PHE319, and L:Comp.14:447 stand out as major contributors to the total energy changes, with values of −2.27, −3.06, and −13.56 kcal/mol, respectively. Conversely, SER320 shows a positive total energy change of 0.55 kcal/mol, primarily driven by polar solvation energy. The remaining residue changes exhibit smaller magnitudes, mostly within 1 kcal/mol, with electrostatic, van der Waals, and polar solvation energies tending to offset each other. The high standard deviations and errors suggest greater variability in these energy terms. In simulations of NOS3 and TX18, the average total change of −1.99 kcal/mol for B:TRP:144 primarily stems from favorable van der Waals and electrostatic interactions, partially counteracted by unfavorable polar solvation energy. The total change of −1.14 kcal/mol for B:ALA:147 is mainly due to favorable van der Waals interactions, somewhat mitigated by electrostatic interactions. For B:ARG:149, the total change is 0.73 kcal/mol, where the favorable polar solvation energy surpasses the combined unfavorable effects of van der Waals and electrostatic interactions. B:CYS:150 exhibits a total change of −0.75 kcal/mol, primarily driven by unfavorable electrostatic interactions, partially compensated by van der Waals interactions and polar solvation energy. The residue B:LEU:159 exhibits a significant total change of −0.63 kcal/mol, primarily driven by a favorable contribution from van der Waals interactions. In contrast, residue B:SER:192 experiences a total change of −0.17 kcal/mol, where the favorable effect of polar solvation energy is counteracted by unfavorable contributions from electrostatic and van der Waals interactions. The total change for residue B:ALA:193 approaches zero at 0.10 kcal/mol, reflecting minimal and nearly balanced energy term contributions. Similarly, residue B:ILE:194 demonstrates a total change of −0.39 kcal/mol, predominantly influenced by the favorable impact of van der Waals interactions. Moreover, residue B:MET:305 displays a modest total change of −0.18 kcal/mol, as the advantageous polar solvation energies counteract the adverse effects of electrostatic and van der Waals interactions. Notably, residue B:SER:320 exhibits a substantial total change of 0.83 kcal/mol, with the beneficial contributions from polar solvation energy and electrostatic interactions surpassing the unfavorable impact of van der Waals interactions. The change in energy for residue B:TYR:441 is minimal at 0.08 kcal/mol, where the polar solvation energy’s favorable effect is counterbalanced by van der Waals and electrostatic interactions. Conversely, the most substantial energy alteration of −17.06 kcal/mol occurs in residue L:Comp.18:447, primarily driven by a significant unfavorable electrostatic interaction, followed by van der Waals forces. Notably, residues TRP144, PHE319, and Comp.18:447 exhibit notable energy fluctuations of −1.99, −2.49, and −17.06 kcal/mol, respectively, predominantly influenced by van der Waals and electrostatic interactions. The majority of residues experience energy changes below 1 kcal/mol, with contributions from various interactions that often offset each other. Some residues, such as ARG149 and SER320, benefit from a minor favorable polar solvation energy contribution (Figure 6E).

### 3.6 Signaling pathway analysis

**FIGURE 7:**
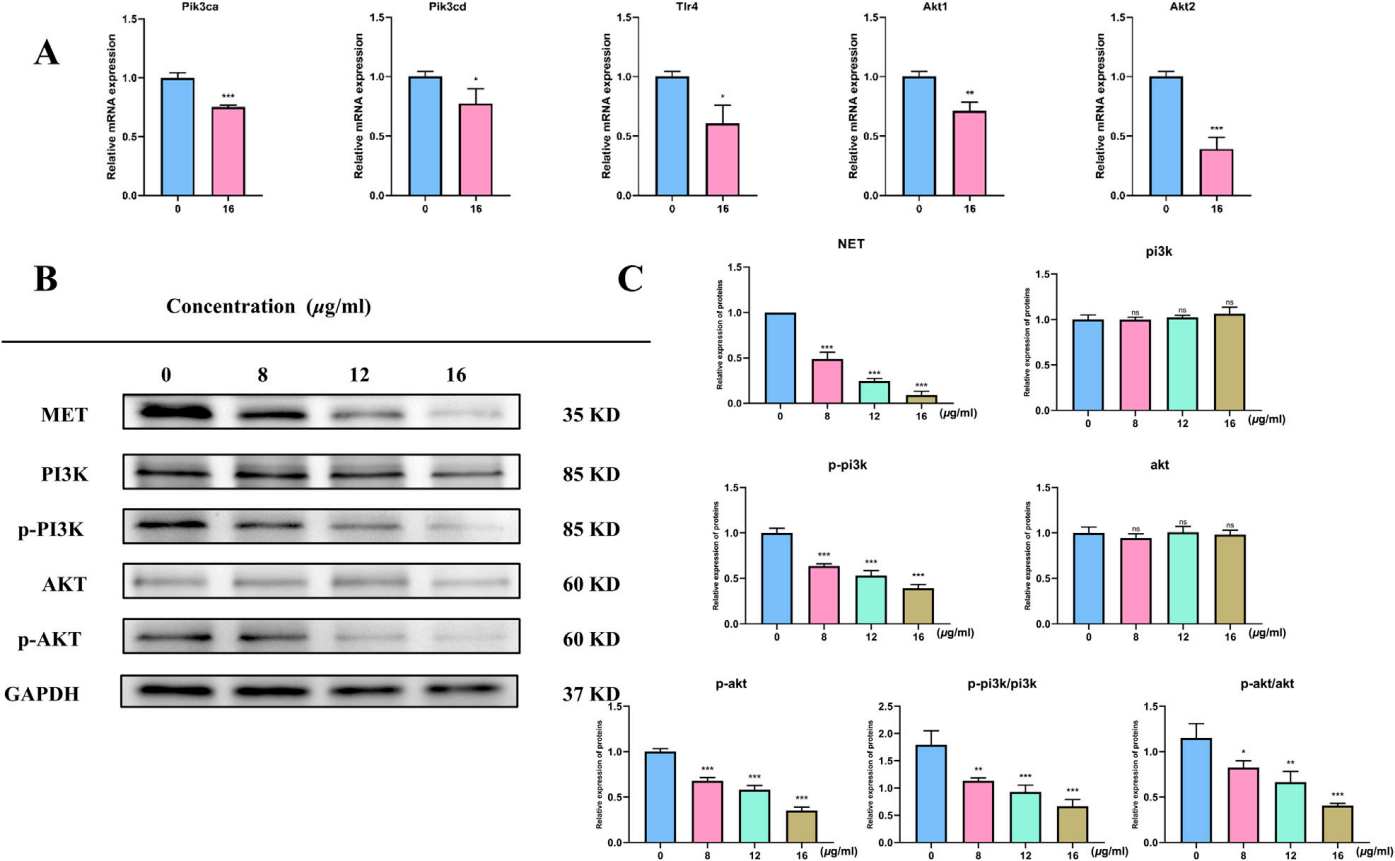
mRNA levels of target gene **(A)** Expression of PIK3/AKT signal pathway protein in RCC cells treated with different concentrations of TX **(B)** Expression of relative expression proteins treated with different concentrations of TX **(C)**. Note: * represents for vs. MOD group (p < 0.05), ** represents for vs. MOD group (p < 0.01), *** represents for vs. MOD group (p < 0.001), respectively.

We employed qRT-PCR to measure the expression of markers linked to the PI3K/AKT pathway and apoptosis following 48 h of treatment with each medication in order to confirm whether TX caused apoptosis in ACHN cells by altering the PI3K/AKT pathway. The mRNA expression of the PIK3CA, PIK3CD, TLR4, AKT1, and AKT2 genes was lower (*P* < 0.05) in the 16 μg/mL TX-treated group than in the MOD group (Figure 7A). In line with KEGG analysis, transcription analysis results showed that the PI3K-Akt pathway was significantly enriched. Both cell division and apoptosis depend on the PI3K/AKT pathway, which is frequently seen to be activated in a variety of cancer cells. The development of RCC is thought to be intimately associated with the activation of the PI3K/AKT pathway. An important component of this signaling pathway, activated AKT greatly aids in the promotion of cell division, growth, and invasive activity. We evaluated the activity of pathway protein level analysis to gain a better understanding of how TX suppresses PI3K/AKT signaling in ACHN cell metastasis. The results showed that TX treatment had no effect on PI3K and AKT gene expression in ACHN cells. However, MET, *p*-PI3K, *p*-PI3K/PI3K, *p*-AKT, and *p*-AKT/AKT were all markedly downregulated at the protein level (Figures 7B,C).

## 4 Discussion

*Trigonostemon xyphophyllorides*, a plant species endemic to Hainan, China, belongs to the genus *Trigonostemon* in the Euphorbiaceae family. It is utilized in traditional medicine for treating snake bites in Thailand and asthma in southern China. Recent studies have revealed additional therapeutic properties of this plant, including anti-HIV, insect repellent, anti-cancer, and antioxidant effects (Ban et al., 2020; Liu et al., 2018a; Liu et al., 2018b). Notably, the active diterpenoids exhibit anti-proliferative activity, although existing reports primarily focus on the IC_50_ values of individual compounds without comprehensive investigation. Therefore, this study selected RCC as a target for pharmacological research due to its established research foundation. Initially, the components of *Trigonostemon* extract were identified using UPLC-Q-TOF-MS/MS. Molecular docking and dynamics simulations were conducted to assess the interaction between components, targets, and the PI3K-AKT signaling pathway, suggesting a potential impact of TX on RCC via this pathway. This hypothesis was further validated through *in vitro* cell experiments, invasion assays, qPCR, and Western blot analysis.

The complexity and diverse mechanisms of traditional Chinese medicine have historically hindered its effective application in modern contexts. To address this challenge, we utilized a combination of UPLC-Q-TOF-MS/MS and network analysis in this study to elucidate the mechanism of action of a specific medicinal herb. This approach aimed to streamline the investigation process and avoid aimless exploration. Molecular docking and dynamics simulations confirmed the strong binding affinity and stable interactions between resveratrol and these core proteins, findings further validated through *in vitro* experiments (Infante Hernández et al., 2024). Transcription analysis not only enables the prediction of individual herb effects but also facilitates the analysis of formulations. By applying transcription analysis to study the Shexiang Baoxin pill (SBP) in atherosclerosis treatment, key target genes (MAPK3, AKT1, and STAT3) associated with the prescription’s efficacy were identified. These genes were also linked to the crucial metabolite trimethylamine-N-oxide (TMAO). *In vivo* experiments demonstrated SBP’s inhibition of TMAO-induced endothelial cell apoptosis and oxidative stress, along with its ability to counteract the upregulation of MAPK3, AKT1, and STAT3 expression (Li D. et al., 2024). Thus, transcription analysis significantly contributes to unraveling the mechanisms underlying modern traditional Chinese medicine and its formulations. Nevertheless, transcription analysis has limitations. Notably, pathway enrichment relies on established mechanisms, potentially limiting the discovery of novel pharmacological principles. In this study, leveraging transcription analysis prediction technology, *in vitro* cell experiments confirmed that TX exerts its therapeutic effects via the PI3K-AKT signaling pathway.

The PI3K/Akt signaling pathway is a crucial intracellular signaling cascade that regulates cell proliferation, apoptosis, and angiogenesis. Upon activation of PI3K, phosphatidylinositol-3,4,5-trisphosphate recruits and activates downstream effectors such as Akt. Furthermore, this pathway can be positively modulated by upstream signaling molecules including growth factor and cytokine receptors. Upon ligand binding, these receptors initiate downstream signaling cascades involving PI3K and Akt, leading to the activation of glycogen synthase kinase-3 (GSK3β), suppression of nuclear factor-κB (NF-κB) and forkhead box protein O1 (FOXO1) transcriptional activities, and promotion of P53 ubiquitination and degradation by murine double minute 2 (MDM2), thereby influencing cell apoptosis. The PI3K/Akt pathway is implicated in RCC treatment, where naringenin has been identified as a potent inhibitor of RCC cell proliferation by modulating Ki67 expression, inducing G2 cell cycle arrest, upregulating caspase-8, downregulating Bcl-2, enhancing PTEN expression to inhibit proliferation and induce apoptosis, and downregulating PI3K and p-AKT (Li L. et al., 2024). Moreover, the pathway is associated with drug resistance, as evidenced by the synergistic effect of resveratrol liposomes (RES-lips) and sorafenib in combating sorafenib-resistant RCC models. This combination treatment leads to G1/S phase arrest in drug-resistant cells, increased tumor growth inhibition (TGI) rate, and complete remission (CR) rate by targeting the PI3K-AKT-mTOR and VHL-HIF pathways to overcome sorafenib resistance (Wang et al., 2024). Despite these findings, limitations exist in the study, such as the identification of endoplasmic reticulum-related enrichment during GO functional analysis, warranting further investigation into the involvement of endoplasmic reticulum stress in RCC treatment. Additionally, the study primarily relies on *in vitro* cell experiments, necessitating validation through *in vivo* animal studies to enhance the comprehensive understanding of the therapeutic mechanisms of the PI3K/Akt pathway in RCC treatment and establish a robust foundation for clinical translation.

## 5 Conclusion

This study utilized *in vitro* experimental validation techniques to establish that TX exerts its anti-proliferative effects by activating the PI3K-AKT signaling pathway. These findings elucidate the mechanism of TX action and provide a scientific basis for its prospective contemporary utilization.

## Data Availability

The original contributions presented in the study are included in the article/Supplementary Material, further inquiries can be directed to the corresponding authors.

## Glossary

AO: acridine orange
BC: betweenness centrality
BP: biological process
CBX3: Chromobox homologue 3
CC: cellular component
CR: complete remission
DEGs: differentially expressed genes
EB: ethidium bromide
ER: endoplasmic reticulum
FBMN: eature-Based Molecular Networking
FBS: fetal bovine serum
FC: fold change
FOXO1: forkhead box protein O1
FPKM: fragments per kilobase of transcript per million fragments mapped
GBSA: generalized Born surface area
GO: Gene Ontology
GSK3β: glycogen synthase kinase-3
H-Bond: hydrogen bonding
HRE: *Hemsleya amabilis* Diels
**IC**_**50**_: Half maximal inhibitory concentration
IVDD: intervertebral disc degeneration
KEGG: Kyoto Encyclopedia of Genes and Genomes
MF: molecular function
MM: molecular mechanics
MMP: matrix metalloproteinase
NAP: Network Annotation Propagation
NF-κB: nuclear factor-κB
OS: overall survival
PME: Particel-Mesh Ewald
PPI: Protein-Protein Interaction
qRT-PCR: Quantitative real-time reverse transcription polymerase chain reaction
RCC: renal cell carcinoma
RG: radius of gyration
RMSD: root mean square deviation
RMSF: root mean square fluctuation
SBP: Shexiang Baoxin pill
TCM: Traditional Chinese medicine
TFE3: transcription factor E3
TFEB: transcription factor EB
TGI: tumor growth inhibition
TMAO: trimethylamine-N-oxide
TPA: 12-O-tetradecanoylphorbol-13-acetate
TX: *Trigonostemon xyphophyllorides.*
