# Supplementary material for: Antiproliferative effects of Trigonostemon xyphophyllorides on renal cell carcinoma via the PI3K/AKT pathway
Source: Front Pharmacol. 2025 Nov 7;16:1594461. doi: 10.3389/fphar.2025.1594461 (PMC12635619; doi:10.3389/fphar.2025.1594461)
Supplement: Supplementary file 1 [file DataSheet1.docx]

Supplementing information

| **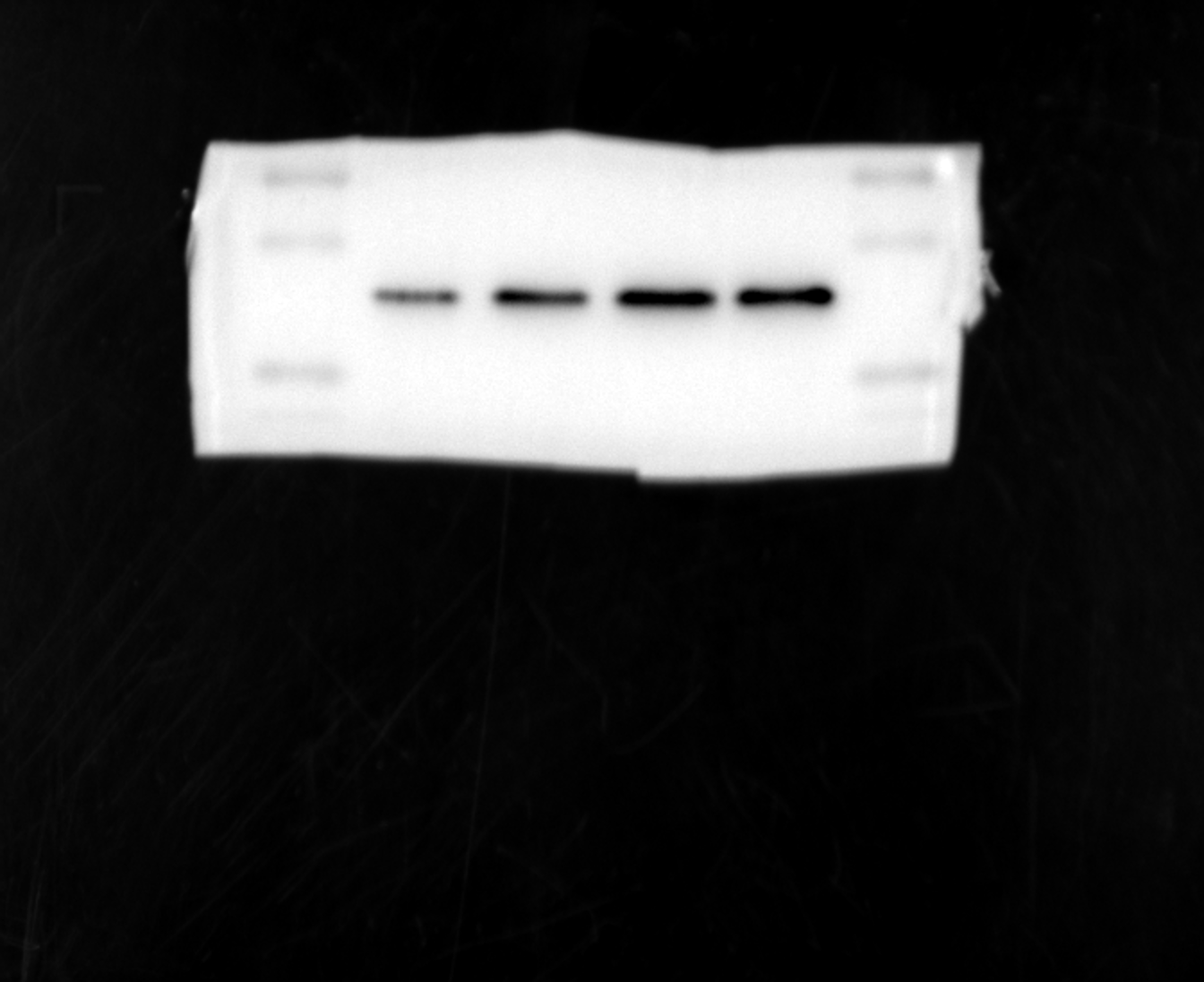** |
| --- |
| Figure S1 Western blot on Bax with varying concentrations of TX |

| **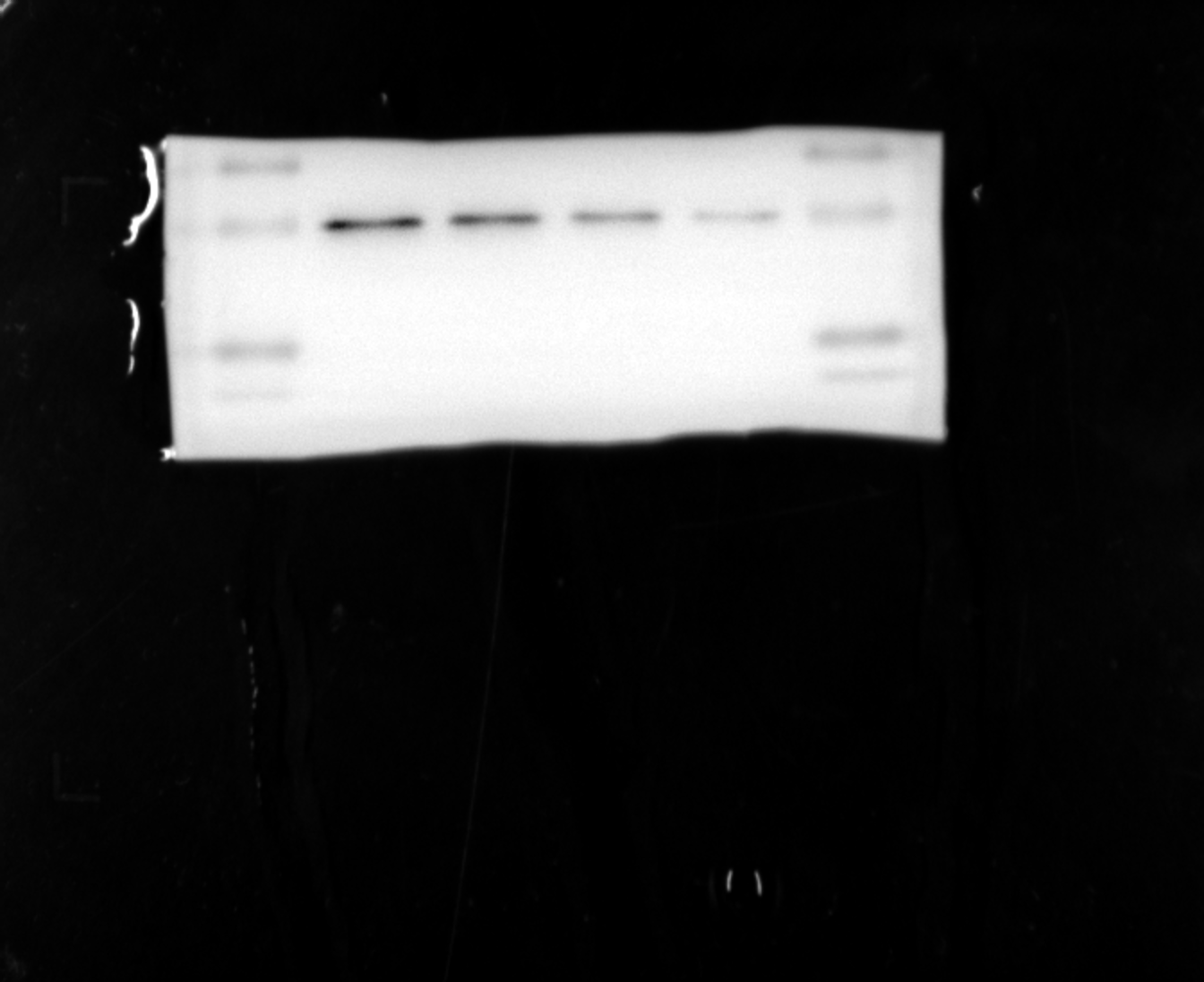** |
| --- |
| Figure S2 Western blot on Bcl-2 with varying concentrations of TX |


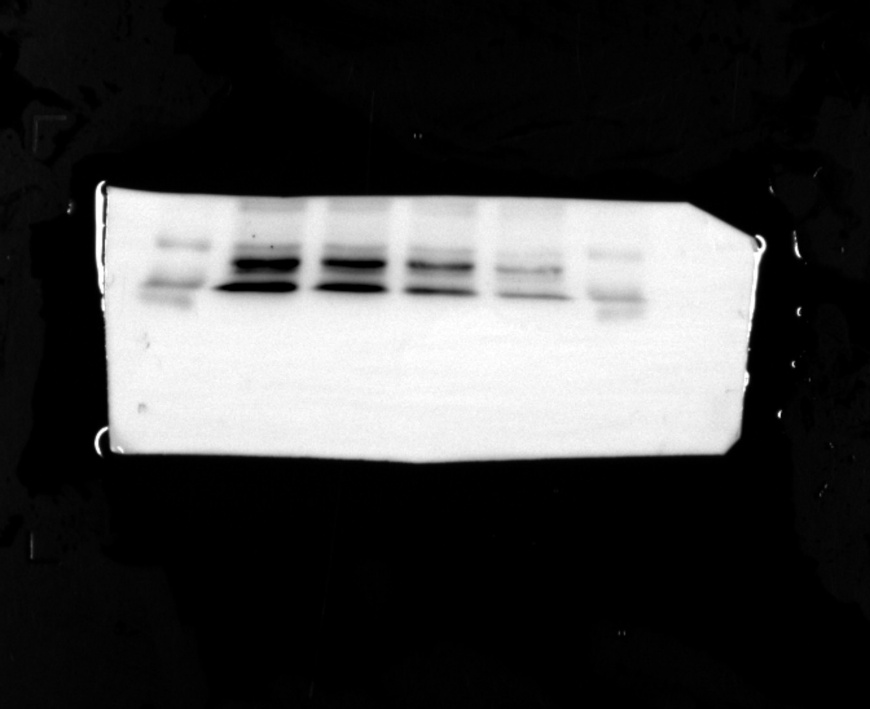


Figure S3 Western blot on Cleaved Caspase 3 with varying concentrations of TX

**
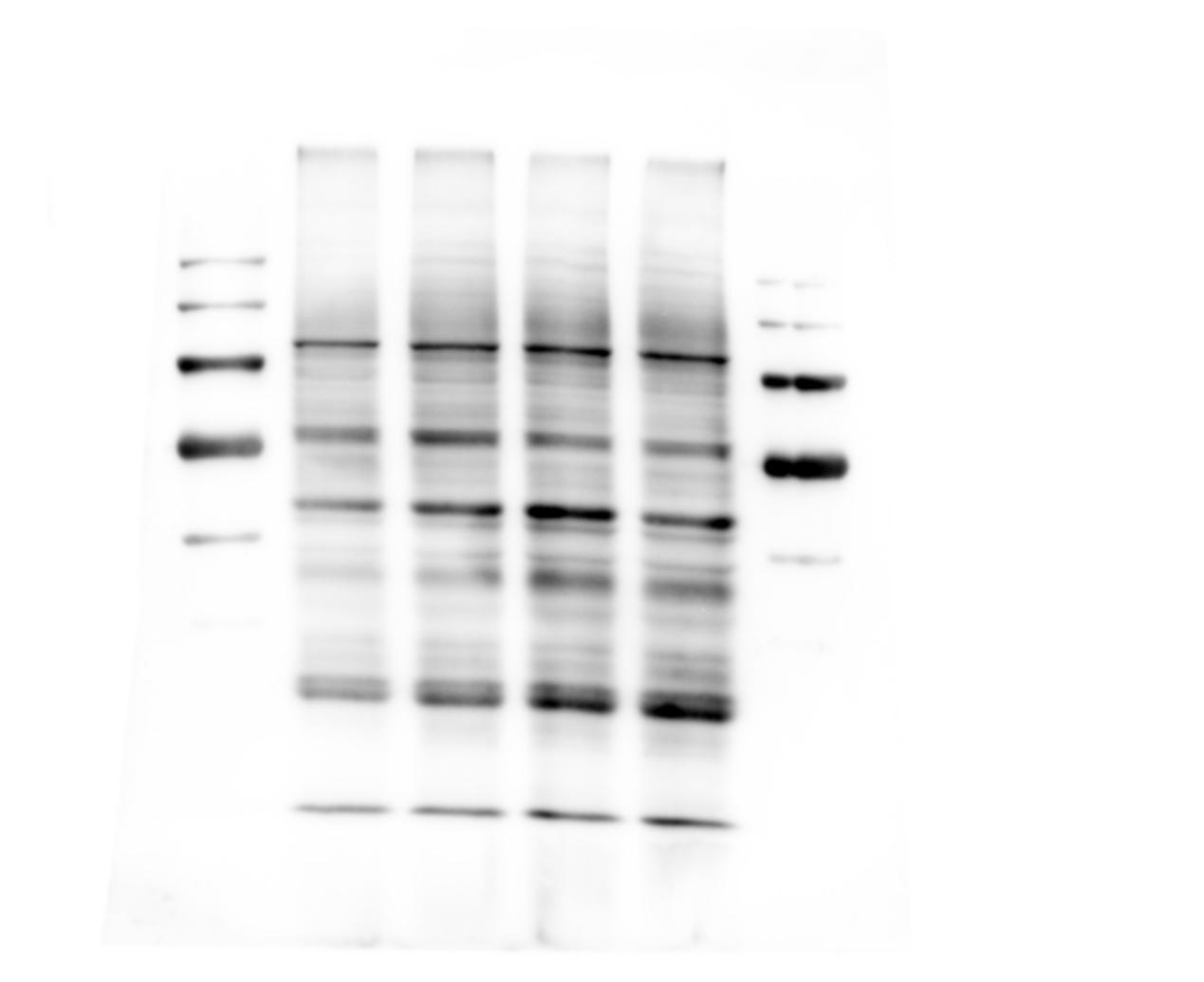
**

|  |
| --- |
| Figure S4 Western blot on PI3K with varying concentrations of TX |

**

**

|  |
| --- |
| Figure S5 Western blot on p-PI3K with varying concentrations of TX |

| **** |
| --- |
| Figure S6 Western blot on AKT with varying concentrations of TX |

**

**

|  |
| --- |
| Figure S7 Western blot on P-AKT with varying concentrations of TX |

**
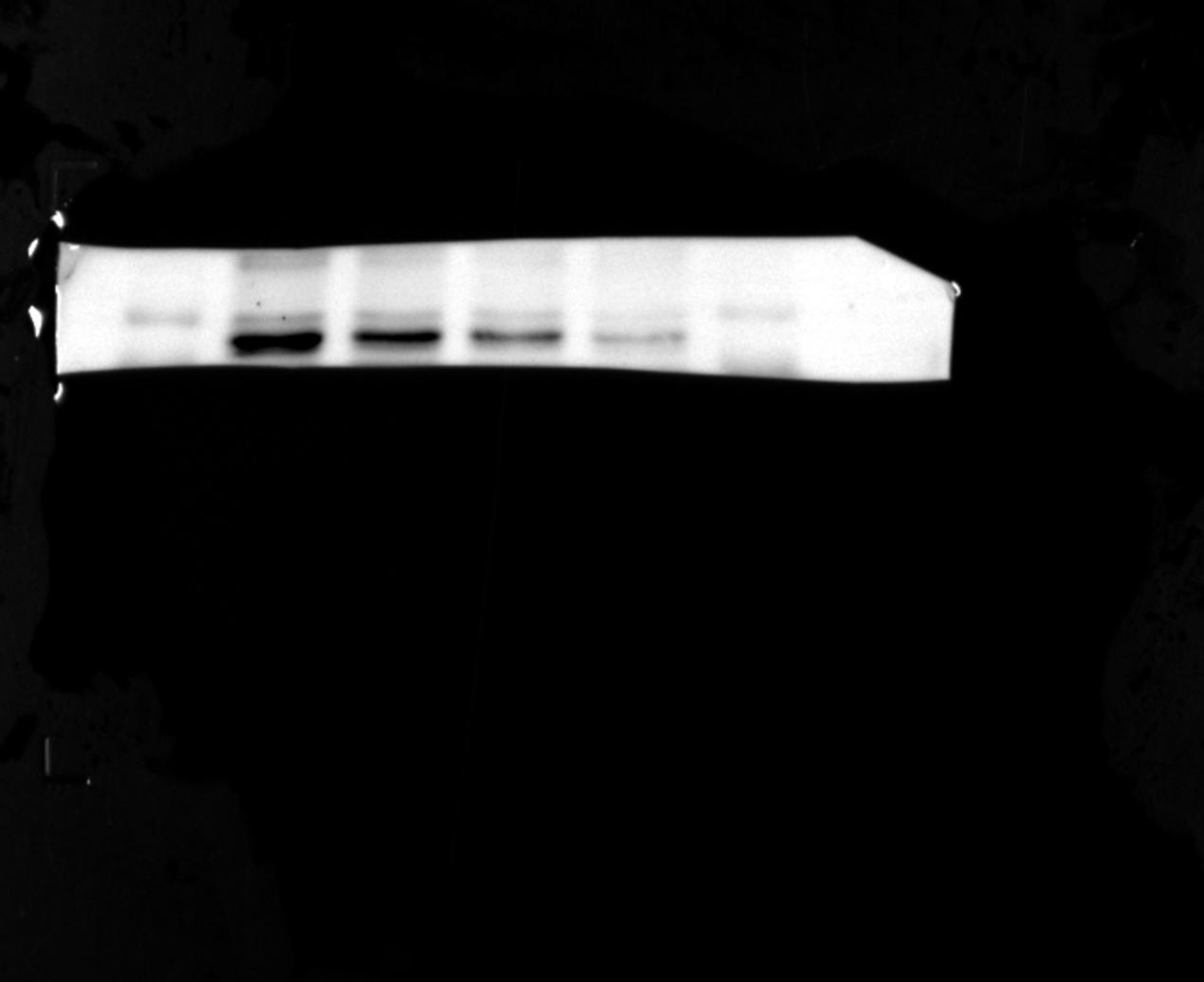
**

Figure S8 Western blot on C-MET with varying concentrations of TX

**

**

Figure S9 Western blot on GAPDH with varying concentrations of TX

**

**

Figure S10 Western blot on GAPDH with varying concentrations of TX
